# Elevated Neutrophil to Lymphocyte Ratio Predicts Poor Prognosis in Non-muscle Invasive Bladder Cancer Patients: Initial Intravesical Bacillus Calmette-Guerin Treatment After Transurethral Resection of Bladder Tumor Setting

**DOI:** 10.3389/fonc.2018.00642

**Published:** 2019-01-17

**Authors:** Hyeong Dong Yuk, Chang Wook Jeong, Cheol Kwak, Hyeon Hoe Kim, Ja Hyeon Ku

**Affiliations:** ^1^Department of Urology, Inje University Sanggye Paik Hospital, Seoul, South Korea; ^2^Department of Urology, Seoul National University Hospital, Seoul, South Korea

**Keywords:** BCG, bladder cancer, neutrophil-to-lymphocyte ratio, systemic inflammatory response, non-muscle invasive bladder cancer

## Abstract

The objective of this study was to investigate pretreatment systemic inflammatory response (SIR) markers in patients who underwent initial intravesical treatment for high-risk non-muscle invasive bladder cancer (NMIBC). A total of 385 patients who underwent initial intravesical Bacillus Calmette-Guerin treatment after transurethral resection of bladder tumor (TURB) were included. We analyzed the relationship between oncological outcomes and ratios of SIR markers, including neutrophil-to-lymphocyte ratio (NLR), derived neutrophil-to-lymphocyte ratio (dNLR), and platelet-to-lymphocyte ratio (PLR). Each SIR marker was used for analysis. Their cut-off values were determined through receiver operation characteristics curves analysis. Patients were divided into two groups according to pretreatment NLR (<1.5 vs. ≥1.5), dNLR (<1.2 vs. ≥1.2), and PLR values (171< vs. ≥171). Patients with NLR ≥ 1.5 and dNLR ≥ 1.2 were associated with poor prognosis in terms of overall survival and cause-specific survival. However, no serum SIR marker was associated with prognosis in recurrence-free survival or progression-free survival. Cox multivariate analysis revealed that age, NLR, dNLR, hemoglobin, and pathologic T stage were significant factors predicting overall survival. Age, NLR, and pathologic T stage were significant factors predicting cancer-specific survival, NLR and tumor number were the most important predictors of bladder preserving survival. NLR before treatment was correlated with both oncological outcomes and survival outcome in NMIBC patients undergoing initial intravesical BCG treatment after TURB. Increased NLR reflects a poor prognosis of these outcomes.

## Introduction

Bladder cancer is the 9th most commonly diagnosed cancer worldwide and the 13th most frequent cause of cancer death worldwide (1). It is also the 5th most common cancer in the United States. Bladder cancer is the most expensive cancer to treat (2).

Most bladder cancers are histologically diagnosed as urothelial cell carcinoma (UC). Ninety percent of UC cases originate in the bladder. Of these, 75% are diagnosed as non-muscle invasive bladder cancer (NMIBC) with mucosal or submucosal confined disease (3, 4). NMIBC has a wide range of tumor biology and heterogeneity (5). Because of this heterogeneity, there are various treatment options. It is difficult to determine standard treatment guidelines (5). Thus, several institutions have performed risk stratification based on the risk of recurrence and progression of tumors (5). Such stratification of risk groups based on recurrence and progression risk of tumors was a strategy used in an attempt to individualize and standardize treatment for specific patient populations (5). The European Association of Urology (EAU) and National Comprehensive Cancer Network (NCCN) guidelines are the two well-known treatment guidelines for this stratification (3, 6). The EAU guidelines defined NMIBC high risk group as T1 tumor, high grade tumor, carcinoma *in situ* (CIS), and Ta low grade tumor with multiple, recurrent, and large (>3 cm) tumor (3). The NCCN guidelines defined NMIBC high risk group as T1 tumor, high grade tumor, CIS (6). Patients whose NMIBC is high grade, T1, or carcinoma *in situ* (CIS) are at high risk of recurrence and progression (1). The standard treatment for NMIBC is transurethral resection of bladder tumor (TURB). However, the recurrence rate is as high as 80% within 5 years after initial TURB (1, 3). In addition, 30% of patients undergo radical cystectomy (3, 5). Most studies on SIR markers in bladder cancer have been focused on treatment for MIBC (7–13). These studies suggest that SIR markers may be useful for predicting tumor prognosis before treatment (7–13). Pretreatment patient-related factors may help predict the prognosis of cancer patients (14). In bladder cancer, factors associated with pretreatment may affect various treatments. They might affect treatment outcome of patients with NMIBC or MIBC. We hypothesized that pretreatment SIR markers in NMIBC patients could affect treatment outcome and prognosis of patients.

To prevent recurrence and rapid progression, we performed intravesical Bacillus Calmette-Guerin (BCG) therapy. In 1976, Morales et al. confirmed the efficacy of BCG therapy for bladder cancer patients and then for NMIBC patients (3, 6). Intravesical BCG treatment is now the standard treatment for high-risk NMIBC patients (3, 6, 15). It is important to determine predictors for clinical outcome to improve BCG treatment decisions in these patients. Therefore, the objective of this study was to investigate associations between pretreatment systemic inflammatory response biomarkers and oncologic outcome of patients with UC.

## Patients and Methods

### Study Sample

The Institutional Review Board of Seoul National University Hospital Clinical Research Institute approved this study (approval code: H-1710-033-891). This was a retrospective study. It was exempt from the requirement of patient written consent. The study protocol and all related items followed the Declaration of Helsinki guidelines. We retrospectively reviewed medical records of 3,127 patients who received TURB at Seoul National University Hospital from 1991 to 2015. Patients who underwent initial intravesical BCG treatment after TURB were included. There was a difference in treatment duration from 1991 to 2015. Indication and recommended method of intravesical BCG treatment also differed for different ages. Therefore, we retrospectively included patients who underwent intravesical BCG treatment among patients who met our defined high-risk group criteria. All patients were high-risk NMIBC patients with T1, high-grade tumor, or CIS. Patients without UC, with short follow-up (<2 years), and those who were suspected of infections accompanied by fever or leukocytosis were excluded. Finally, 385 patients were included.

### Study Design

Pretreatment systemic inflammatory response markers were investigated. We collected clinicopathological information from patient's medical records at the time of initial intravesical treatment after TURB. Clinicopathological information included age, sex, body mass index (BMI), hypertension, diabetes, lung disease, absolute neutrophil, lymphocyte, and platelet counts, hemoglobin, pre-intravesical treatment NLR, dNLR, and PLR values, creatinine, estimated glomerular filtration ratio (eGFR), pathological T stage, tumor grade, CIS, lymphovascular invasion (LVI), tumor number, and tumor size. We also collected various oncologic prognoses and outcomes, including upper urinary tract recurrence, recurrence in bladder, history and timing of radical cystectomy, progression, cancer-specific mortality, and overall mortality. SIR markers calculated included neutrophil-to-lymphocyte ratio (NLR, absolute neutrophil count/lymphocyte count), derived neutrophil-to-lymphocyte ratio [dNLR (absolute neutrophil count/white blood cell count) – neutrophil count], and platelet-to-lymphocyte ratio (PLR, absolute platelet count/lymphocyte count).

We performed TURB and intravesical treatment according to established principles of our hospital. Specimens resected by TURB were histologically examined and reviewed by two experienced urological pathologists. They determined the TNM stage and tumor grade according to the 2010 American Joint Committee on Cancer classification and the 2004 World Health Organization/International Society of Urologic Pathology consensus classifications principles. Almost all patients were followed-up every 3 months for 3 years first. They were then followed up every 6 months for 2 more years (year 5) and annually thereafter. At each follow-up, basic laboratory tests, urine analysis, urine cytology, and cystoscopy were performed. Computed tomography (CT) was performed annually to check progression and upper tract recurrence. Intravesical BCG treatment was performed. The BCG schedule was as follows: BCG induction therapy (weekly BCG instillation for the first 6 weeks) and maintenance therapy for up to 3 years (3 weekly maintenance instillation for 3, 6, 12, 18, 24, 30, and 36 months).

### Statistical Analyses

The primary end point was overall survival (OS). Secondary end points were recurrence-free survival (RFS), progression-free survival (PFS), bladder preserving survival (BPS), and cancer-specific survival (CSS). Kaplan-Meier survival analysis and log rank test were used for all survival analyses. Cox proportional hazard regression analysis was used to analyze various independent predictors of oncological outcomes. Univariate analysis was used for various factors related to patient characteristics and tumor characteristics before initial intravesical BCG treatment. Multivariate analysis was performed only for variables with *P* < 0.1 in univariate analysis. By identifying explanatory variables that were significantly related to response variable, dozens of variables were considered one at a time. A less restrictive level (*P* < 0.1) is often used to identify many explanatory variables that can be associated with response variables in univariate analysis. Thus, we used a limited number of variables with *P* < 0.1 in the univariate analysis for the multiple regression model. We used receiver-operating characteristic (ROC) curve to find appropriate cut off values for SIR markers. The optimal cut-off value was selected as the point at which the sensitivity and specificity were maximized for the prediction of oncologic outcomes. In the ROC curve analysis (AUC = 0.623; 95% CI = 0.55–0.69; *p* < 0.001), dNLR (AUC = 0.592; 95% CI = 0.52–0.67; *p* < 0.001), and PLR (AUC = 0.674; 95% CI = 0.50–0.64; *p* < 0.001) showed predictive power to distinguish OS. Continuous variables are presented as mean values with standard deviation (SD) while categorical variables are presented as ratio of events (%). Statistical significance was defined at *p* ≤ 0.05. IBM SPSS Statistics version 22.0 (IBM, Armonk, NY, USA) was used for all statistical analysis.

## Results

### Clinicopathological Characteristics of Patients Who Underwent Initial Intravesical BCG Treatment After TURB

A total of 385 high risk NMIBC cases were diagnosed after TURB and initial intravesical BCG treatment was performed. The mean follow-up time was 80 months [Standard deviation ± 50.7 months]. Most (84.9%) patients were males. Nearly half (48.3%) of these patients experienced recurrence and 11.4% of patients had disease progression. Radical cystectomy was performed for 13.2% of patients. The overall mortality rate was 18.2%. Cancer-specific mortality rate was 4.7%. The mean time to recurrence was 43 months while the mean time to progression was 31 months. Median values of SIR markers were: NLR, 1.86 (IQR: 0.26–3.50); dNLR, 1.36 (IQR: 0.22–3.09); and PLR, 113.25: (IQR 35.3–200.8) (Table 1).

**Table 1:** Clinicopathological characteristics of NMIBC patients who underwent initial intravesical BCG treatment after TURB.

**Variables**	**Total (*N* = 385)**
Age	72.6 ± 10.6
**SEX**	
Man	327 (84.9%)
Woman	58 (15.1%)
BMI	24.2 ± 3.1
Hypertension	150 (39.0%)
Diabetes	70 (18.2%)
Lung disease	30 (7.8%)
White blood cell	6.5 ± 1.7
Platelet	218.9 ± 56.7
NLR	2.2 ± 1.2
dNLR	1.5 ± 0.7
PLR	122.6 ± 50.6
Hemoglobin	14.1 ± 2.2
Serum creatinine	1.1 ± 0.4
eGFR	73.0 ± 17.5
**pT STAGE**	
Tis	47 (12.2%)
Ta	86 (22.3%)
T1	252 (65.5%)
**TUMOR GRADE**	
None	9 (2.3%)
Low grade	50 (13.0%)
High grade	326 (84.7%)
Concurrent CIS	105 (27.3%)
LVI	8 (2.1%)
**TUMOR NUMBER**	
1	148 (38.4%)
2–7	186 (48.3%)
≧3	51 (13.2%)
**TUMOR SIZE**	
<3 cm	290 (75.3%)
≧3 cm	95 (24.7%)
Radical cystectomy (N/Person-Years)	51 (13.2%)/84.8
Upper urinary tract recurrence (N/Person-Years)	16 (4.2%)/72.1
Recurrence (N/Person-Years)	186 (48.3%)/669.3
Progression (N/Person-Years)	44 (11.4%)/116.1
Cancer specific mortality (N/Person-Years)	18 (4.7%)/103.6
Total mortality (N/Person-Years)	70 (18.2%)/300.6

*NMIBC, non-muscle invasive bladder cancer; BMI, body mass index; SIR, systemic inflammatory response; NLR, neutrophil-to-lymphocyte ratio; dNLR, derived NLR; PLR, platelet-lymphocyte ratio; CIS, carcinoma in situ*.

### Correlation of Serum SIR Markers With Oncologic and Survival Outcomes

**Figure 1:**
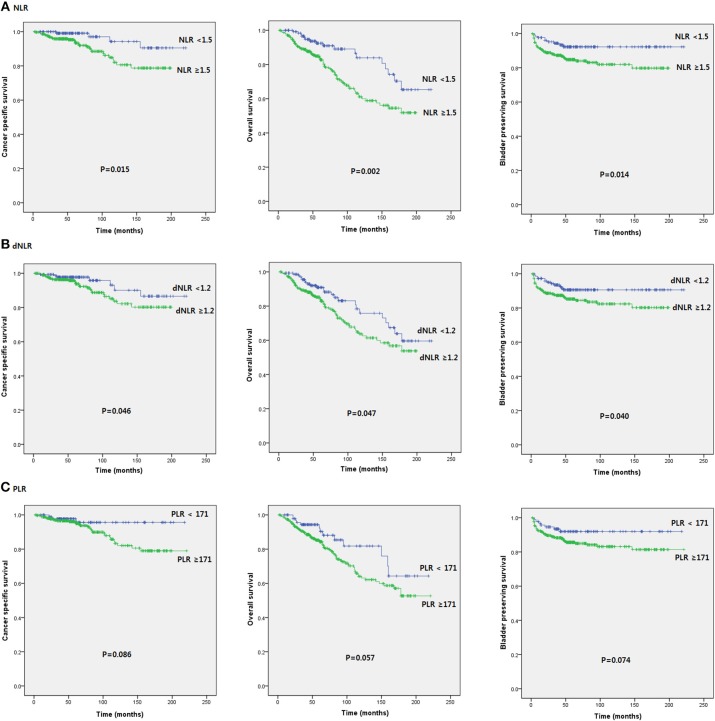
Kaplan-Meier survival curves of oncological outcomes according to pretreatment SIR markers in initial BCG patients after TURB.

Patients were divided into two groups according to pretreatment NLR (<1.5 vs. ≥1.5), dNLR (<1.2 vs. ≥1.2), and PLR values (171< vs. ≥171). Patients with NLR ≥ 1.5 and dNLR ≥ 1.2 were associated with poor prognosis in terms of OS (*p* = 0.002 and *p* = 0.047, respectively), CSS (*p* = 0.015 and *p* = 0.046, respectively), and BPS (*p* = 0.014 and *p* = 0.040, respectively). However, no serum SIR marker was associated with prognosis in terms of RFS or PFS. Unexpectedly, PLR did not help predict any oncologic or survival outcome in patients with intravesical BCG treatment (Figure 1).

### Important Predictors of Oncological Outcomes by Cox Multivariate Regression Analysis

Important predictors of OS in multivariate analysis were age, NLR, dNLR, Hb, and pathologic T stage (Table 2). Important predictors of CSS were age, NLR, and pathologic T stage (Table 3). NLR and tumor number were the most important predictors for BPS (Table 4). ROC curve analysis showed that NLR and dNLR had predictive power for OS of patients with TURB after initial intravesical BCG treatment (area under the curve [AUC] = 0.59; 95% CI = 0.52–0.65; *p* < 0.001 and AUC = 0.55; 95% CI = 0.49–0.62; *p* < 0.001).

**Table 2:** Multivariate Cox proportional hazard ratio analysis to identify predictive factors for overall survival.

**Parameter**	**Univariate**		**Multivariate**	
	**HR (95% Cl)**	***P*-value**	**HR (95% Cl)**	***P*-value**
Age	1.11 (1.07–1.15)	0.000	1.08 (1.05–1.10)	0.000
**NLR**				
NLR < 1.5	Reference		Reference
NLR ≥ 1.5	1.94 (0.82–4.58)	0.012	2.24 (1.26–3.96)	0.005
**dNLR**				
dNLR < 1.2	Reference		Reference
dNLR ≥ 1.2	2.12 (1.16–4.11)	0.010	1.65 (0.98–2.55)	0.050
**PLR**				
PLR < 171	Reference		Reference
PLR > 171	0.92 (0.46–1.82)	0.819	0.74 (0.39–1.39)	0.357
Hb	0.82 (0.72–0.94)	0.005	0.80 (0.68–0.95)	0.011
**T STAGE**				
<T1	Reference		Reference
T1	1.98 (1.19–3.30)	0.008	1.99 (1.09–3.64)	0.025
**TUMOR GRADE**				
Low	Reference		Reference
High	2.15 (0.88–5.24)	0.089	1.76 (0.73–4.25)	0.209
Concomitant CIS	1.65 (0.93–2.93)	0.089	1.95 (0.98–3.86)	0.054
LVI	4.71 (1.15–19.32)	0.031	1.67 (0.59–4.73)	0.335
Tumor Number		0.264		0.780
1	Reference		Reference
2~7	1.57 (0.89–2.78)	0.123	1.21 (0.70–2.04)	0.482
≥3	1.07 (0.44–2.57)	0.888	1.11 (0.48–2.52)	0.809
**TUMOR SIZE**				
<3 cm	Reference		Reference
≥3 cm	1.30 (0.76–2.24)	0.332	1.61 (0.83–3.13)	0.162

*NLR, neutrophil-to-lymphocyte ratio; dNLR, derived NLR; PLR, platelet-lymphocyte ratio; Hb, hemoglobin; CIS, carcinoma in situ; LVI, lymphovascular invasion*.

**Table 3:** Multivariate Cox proportional hazard ratio analysis to identify predictive factors for cancer specific survival.

**Parameter**	**Univariate**		**Multivariate**	
	**HR (95% Cl)**	***P*-value**	**HR (95% Cl)**	***P*-value**
Age	1.18 (1.12–1.25)	0.000	1.10 (1.03–1.16)	0.003
**NLR**				
NLR < 1.5	Reference		Reference
NLR ≥ 1.5	3.17 (1.22–10.85)	0.003	2.03 (1.03–3.99)	0.039
**dNLR**				
dNLR < 1.2	Reference		Reference
dNLR ≥ 1.2	1.95 (0.61–6.16)	0.194	1.25 (0.38–4.13)	0.709
**PLR**				
PLR < 171	Reference		Reference
PLR > 171	2.19 (0.49–9.75)	0.302	1.52 (0.29–7.83)	0.615
Hb	0.94 (0.70.−1.24)	0.939	0.90 (0.65–1.26)	0.555
**T STAGE**				
<T1	Reference		Reference
T1	2.77 (1.42–5.40)	0.003	2.36 (1.52–3.66)	0.005
**TUMOR GRADE**				
Low	Reference		Reference
High	1.11 (0.31–3.96)	0.871	1.45 (0.36–5.83)	0.602
Concomitant CIS	6.72 (0.88–51.16)	0.066	2.43 (0.28–21.23)	0.422
LVI	3.02 (0.35–25.99)	3.025	1.25 (0.16–9.75)	0.832
Tumor Number		0.125		0.543
1	Reference		Reference
2~7	3.63 (1.01–12.99)	0.047	2.05 (0.56–7.52)	0.280
≥3	1.97 (0.32–12.15)	0.464	1.50 (0.22–10.11)	0.675
**TUMOR SIZE**				
<3 cm	Reference		Reference
≥3 cm	5.85 (0.77–44.58)	0.088	4.99 (0.63–39.7)	0.129

*NLR, neutrophil-to-lymphocyte ratio; dNLR, derived NLR; PLR, platelet-lymphocyte ratio; Hb, hemoglobin; CIS, carcinoma in situ; LVI, lymphovascular invasion*.

**Table 4:** Multivariate Cox proportional hazard ratio analysis to identify predictive factors for Bladder preserving survival.

**Parameter**	**Univariate**		**Multivariate**	
	**HR(95% Cl)**	***P*-value**	**HR(95% Cl)**	***P*-value**
Age	1.01 (0.98–1.05)	0.261	0.99 (0.97–1.02)	0.691
**NLR**				
NLR < 1.5	Reference		Reference
NLR ≥ 1.5	3.25 (1.34–7.86)	0.009	2.97 (1.19–7.42)	0.019
**dNLR**				
dNLR < 1.2	Reference		Reference
dNLR ≥ 1.2	1.74 (0.81–3.74)	0.154	1.32 (0.64–2.69)	0.444
**PLR**				
PLR < 171	Reference		Reference
PLR > 171	2.17 (0.89–5.29)	0.088	1.43 (0.58–3.55)	0.439
Hb	0.96 (0.81–1.13)	0.641	0.91 (0.76–1.10)	0.342
**T STAGE**				
<T1	Reference		Reference
T1	1.31 (0.69–2.49)	0.409	1.23 (0.64–2.37)	0.528
**TUMOR GRADE**				
Low	Reference		Reference
High	2.31 (0.80–6.69)	0.121	1.42 (0.63–3.19)	0.389
Concomitant CIS	1.25 (0.63–2.50)	0.520	1.31 (0.65–2.67)	0.446
LVI	1.07 (0.12–8.88)	0.950	1.69 (0.39–7.26)	0.479
Tumor Number		0.48		0.041
1	Reference		Reference
2~7	2.18 (1.07–4.42)	0.031	2.10 (1.04–4.24)	0.038
≥3	2.43 (0.96–6.16)	0.042	2.84 (1.18–6.82)	0.019
**TUMOR SIZE**				
<3 cm	Reference		Reference
≥3 cm	1.89 (0.86–4.18)	0.115	2.11 (0.95–4.67)	0.066

*NLR, neutrophil-to-lymphocyte ratio; dNLR, derived NLR; PLR, platelet-lymphocyte ratio; Hb, hemoglobin; CIS, carcinoma in situ; LVI, lymphovascular invasion*.

### Subgroup Analysis For Patients With Carcinoma *in situ*

**Figure 2:**
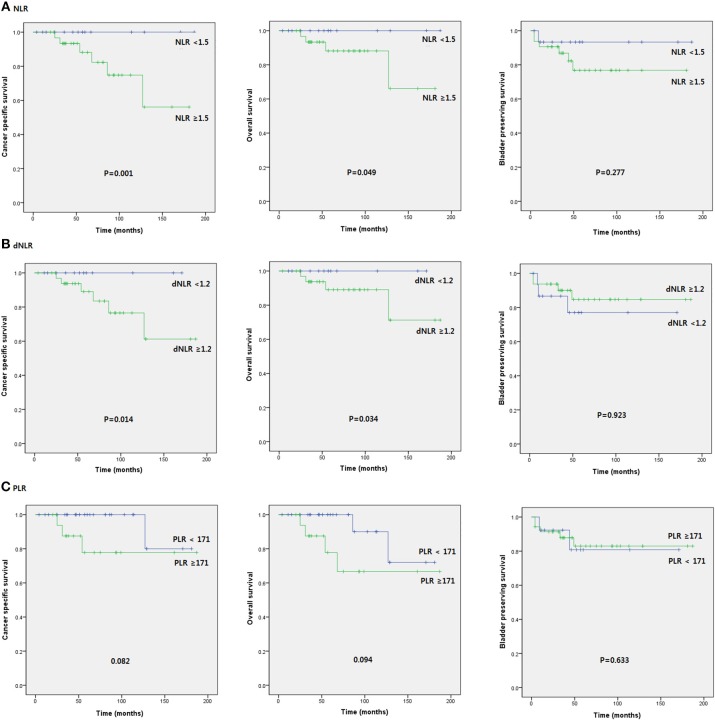
Kaplan-Meier survival curves of oncological outcomes according to pretreatment SIR markers in subgroup of patients with CIS.

In subgroup analysis of CIS patients, patients with NLR ≥ 1.5 and dNLR ≥ 1.2 had poor prognosis for OS (*p* = 0.04 and *p* = 0.034, respectively) and CSS (*p* = 0.001 and *p* = 0.014, respectively). However, there was no statistically significant relationship of NLR or dNLR with BPS, PFS, or RFS. PLR was not significantly associated with oncologic survival outcomes (Figure 2).

## Discussion

Cancer is affected by a variety of factors, including tumor-related and patient-related factors. Until recently, tumor-related factors, such as pathologic stage and grade have been used predominantly in predicting prognosis in cancer patients. However, patient-related factors can also predict the prognosis of cancer patients. These include weight loss, performance status, and SIR. Since Virchow first observed the presence of leukocytes in neoplastic tissue, the association of SIR with tumor progression has been demonstrated (14). Tumors can produce an intrinsic inflammatory response and a protumorigenic microenvironment. Increased inflammation in such a microenvironment promotes cancer activity (16). Accelerated cancer activity will act on immune cells, leading to an increase in systemic immune responses (16). Many recent clinical studies support that SIR is a significant predictor of prognosis in various types of cancer, including esophagus, gastric, colorectal, liver, pancreas, breast, ovaries, cervix, prostate, kidney, and bladder cancers (7–13).

There are few studies on the association between SIR markers and NMIBC. Most studies have been performed on muscle invasive bladder cancer (MIBC) and radical cystectomy. These studies suggest that NLR before radical cystectomy may help predict tumor prognosis (17–21). The cut-off value of preoperative NLR ranges from 2 to 3. Increased NLR is an independent predictor of OS, CSS, and RFS (17–21). A recent meta-analysis using 23 studies and ~6,000 MIBC patients has shown that increased NLR is highly relevant to OS, CSS, and RFS (22). In our study, increased NLR was a predictor of OS and CSS. In addition, increased NLR was helpful in predicting BPS. However, unlike previous studies, there was no significant correlation between increased NLR and RFS predictions (Figure 1). In multivariate analysis, age, increased NLR, and pathologic T stage were important predictors of OS and CSS while increased NLR and tumor numbers were important predictors of BPS (Tables 1–3). Cut off values of NLR and dNLR from ROC curve analysis were 1.5 and 1.2, respectively, which were slightly lower than those (between 2 and 3) reported in previous studies (17–21). Such discrepancy might be due to different timing of execution, difference in disease status, differing immune responses of the host, and differences in tumor characteristics. The difference between the current study and prior studies was that NMIBC patients were our study subject and the analysis was concerned about SIR marker before initial BCG treatment in high-risk pathologic patients after TURB, not surgery. Previous studies have shown that increased NLR before TURB in NMIBC patients is a predictor of OS and CSS (23). The cut-off value was 2.0 for NLR and 1.5 for dNLR (23). There may be difference between surgery and intravesical BCG. However, both NMIBC studies showed similar results (23). NLR was associated with survival outcome. It was not significantly associated with oncological outcomes, such as recurrence or progression (23). In another study, preoperative NLR was favorable to predict recurrence and progression in MIBC patients with initial TURB (24). This is different from results of the present study. A subgroup analysis of the adjuvant BCG group in a recently published NMIBC study has shown that high NLR is associated with disease progression, but not disease recurrence (25).

Unlike previous studies, our results showed that pretreatment NLR or dNLR did not increase the presence of tumor grade, number, size, concomitant CIS, or LVI. This may be due to insufficient patient pool. In addition, our study was performed on patients after TURB. In mechanistic view, advanced stage and aggressiveness may be associated with increased host immune response. However, in this study, all patients were NMIBC patients with low tumor burden after TURB. Such patient status seems to be difficult to reflect characteristics of the tumor.

This study has several limitations. First, it was a retrospective study of a single institution. Thus, it cannot be free from selection bias. Second, the timing of pretreatment NLR measurements was not exactly the same. Third, a single test does not represent the entire systemic immune response state. Fourth, values and confidence intervals of the AUC of the ROC curves cannot show solid results to establish these values as cutoff points with sufficient prediction.

In conclusion, NLR before treatment was correlated with both oncological outcomes and survival outcome in NMIBC patients undergoing initial intravesical BCG treatment after TURB. Increased NLR reflects poor prognosis of these outcomes. Although large-scale prospective studies are needed to apply NLR to clinical practice, NLR will be a useful tool to help improve risk assessment and treatment guidelines for intravesical treatment in high-risk NMIBC patients.

## Author Contributions

HY, CJ, HK, and JK contributed to the concept and project planning. HY, CJ, and CK contributed significantly to the writing of this paper. JK contributed significantly to the editing of the final draft.

### Conflict of Interest Statement

The authors declare that the research was conducted in the absence of any commercial or financial relationships that could be construed as a potential conflict of interest.
